# Extensive phenotypic characterization of a new transgenic mouse reveals pleiotropic perturbations in physiology due to mesenchymal *hGH* minigene expression

**DOI:** 10.1038/s41598-017-02581-8

**Published:** 2017-05-25

**Authors:** Aimilios Kaklamanos, Jan Rozman, Manolis Roulis, Niki Karagianni, Maria Armaka, Moya Wu, Laura Brachthäuser, Julia Calzada-Wack, Marion Horsch, Johannes Beckers, Birgit Rathkolb, Thure Adler, Frauke Neff, Eckhard Wolf, Valerie Gailus-Durner, Helmut Fuchs, Martin Hrabe de Angelis, George Kollias

**Affiliations:** 1 Biomedical Sciences Research Center (B.S.R.C.) “Alexander Fleming”, 16672 Vari, Greece; 2 Biomedcode Hellas SA, Vari, Greece; 3 Department of Physiology, Medical School, National and Kapodistrian University of Athens, 11527 Athens, Greece; 4 German Mouse Clinic, Institute of Experimental Genetics, Helmholtz Zentrum München, German Research Center for Environmental Health GmbH, Ingolstädter Landstrasse 1, 85764 Neuherberg, Germany; 5 German Center for Diabetes Research (DZD), Ingolstädter Landstraße 1, 85764 Neuherberg, Germany; 6 Ludwig-Maximilians-Universität München, Gene Center, Institute of Molecular Animal Breeding and Biotechnology, Feodor-Lynen Strasse 25, 81377 Munich, Germany; 7 Institute of Pathology, Helmholtz Zentrum München, German Research Center for Environmental Health, Ingolstädter Landstrasse 1, 85764 Neuherberg, Germany; 8 Chair of Experimental Genetics, Center of Life and Food Sciences Weihenstephan, Technische Universität München, Ingolstaedter Landstrasse 1, 85354 Freising, Weihenstephan Germany; 9 Department of Immunobiology, Yale University School of Medicine, New Haven, CT 06520 USA

## Abstract

The human growth hormone (*hGH*) minigene used for transgene stabilization in mice has been recently identified to be locally expressed in the tissues where transgenes are active and associated with phenotypic alterations. Here we extend these findings by analyzing the effect of the hGH minigene in TgC6hp55 transgenic mice which express the human TNFR1 under the control of the mesenchymal cell-specific CollagenVI promoter. These mice displayed a fully penetrant phenotype characterized by growth enhancement accompanied by perturbations in metabolic, skeletal, histological and other physiological parameters. Notably, this phenotype was independent of TNF-TNFR1 signaling since the genetic ablation of either *Tnf* or *Tradd* did not rescue the phenotype. Further analyses showed that the *hGH* minigene was expressed in several tissues, also leading to increased hGH protein levels in the serum. Pharmacological blockade of GH signaling prevented the development of the phenotype. Our results indicate that the unplanned expression of the *hGH* minigene in CollagenVI expressing mesenchymal cells can lead through local and/or systemic mechanisms to enhanced somatic growth followed by a plethora of primary and/or secondary effects such as hyperphagia, hypermetabolism, disturbed glucose homeostasis, altered hematological parameters, increased bone formation and lipid accumulation in metabolically critical tissues.

## Introduction

Early during the development of transgenic technologies in mice it was realized that homologous and heterologous intronic sequences and polyadenylation signals are essential for the stabilization and efficient expression of the transgene of interest^1, 2^. It was in the early 1990s when scientists, for this exact purpose, started using the so-called *hGH* minigene which consists of the entire human growth hormone coding region, including introns and a polyadenylation signal^3, 4^. At that time and for many years afterwards, it was believed that since the *hGH* minigene was the second cistron in the transgene-encoded mRNA, it would not be expressed. Therefore this technique was widely used in the generation of new transgenic lines^5–10^, especially in mice designed to express the Cre-recombinase. The number of mice that carry the *hGH* minigene nowadays exceeds 200^11^ (indicatively, only the different pancreatic β-cell specific transgenic mice are at least 22 as reported by Brouwers *et al*.^12^).

However, during the last years there has been an increasing number of publications reporting the expression of this minigene with subsequent effects on the mouse phenotype^12–18^. For example the expression of the *hGH* minigene specifically in the pancreatic β-cells of the mouse negatively affects β-cell physiology as well as glucose and insulin homoeostasis^12–14, 17^. Additionally, the expression of the *hGH* minigene in the mouse brain (either in the hypothalamus or in the pituitary) leads to growth defects and metabolic deregulations through its interaction with the endogenous GHRH-GH-IGF1 axis^15, 16, 18^. The finding that hGH can bind to and activate both the mouse GH receptor and the mouse prolactin receptor (PRLR)^19^ adds another level of complexity in the effects of the unplanned *hGH* minigene expression.

Unfortunately though, in the vast majority of the 200 transgenic mice mentioned above, the authors did not examine whether the *hGH* minigene was expressed, and if they did, they did not examine whether the produced hGH was functional^8, 20–22^. Furthermore, the publications that described the production and functionality of the hGH (coming from the *hGH* minigene) examined only the local effects at the tissue-site of expression and did not assess the possible systemic effects of circulating hGH occurring through less predictable mechanisms^12–18^. Therefore, the ‘safety’ and/or suitability of using mice containing this specific minigene is still debatable since only now we have started discovering the pitfalls related with this technique which has been in use for more than 20 years.

We have previously shown that TNF signaling through its receptor 1 (TNFR1) in the mesenchyme is sufficient to drive pathogenesis in mouse models of Rheumatoid Arthritis and Crohn’s-like Inflammatory Bowel Disease^23^. From the two receptors of TNF, named TNFR1 and TNFR2, human TNFR1 can recognize mouse TNF and vice versa, while this is not the case for TNFR2^24^. In order to study the role of TNF-TNFR1 signaling in the mesenchyme in a humanized background, we generated the TgC6hp55 transgenic mice which, additionally to the mouse *Tnfr1*, express the human TNFR1 specifically in the mesenchymal compartment under the control of the Col6a1 (simply CollagenVI or ColVI) promoter^25^. It has been previously shown in transgenic embryos that the CollagenVI promoter is active in mesenchymal cells in many different tissues like skin insertions of the superficial aponeurosis, joints, nerves, intervertebral discs, vibrissae, skeletal muscle, meninges, tendons, subepidermal mesenchyme, heart, blood vessels, adipose tissue, cartilage, central nervous system and retina (ectopic expression), while there are some hints for expression in other tissues like lungs, intestine, kidney, periosteum, perichondrium, serosae, bladder^25^.

Here we show that the expression of the *hGH* minigene in the CollagenVI expressing mesenchymal cells in various tissues can lead to previously undescribed alterations in mouse physiology. The extensive phenotypic characterization of the transgenic mice TgC6hp55 discovered no signs of spontaneous inflammation and/or autoimmunity but revealed a variety of perturbations in many different aspects of their physiology including somatic growth, skeletal characteristics, as well as histological alterations in the liver and adipose tissue that suggested enhanced lipid storage. Many findings resemble effects of transgenic systemic growth hormone overexpression. Our molecular analysis uncovered the expression of the *hGH* minigene in different tissues and our pharmacological experiments confirmed the causal relation between the produced hGH and the observed phenotype. Our findings describe for the first time the plethora of local and/or systemic effects that the *hGH* minigene can have when expressed in the mesenchymal compartment.

## Results

### Generation of the TgC6hp55 transgenic mice and assessment of transgene expression

**Figure 1:**
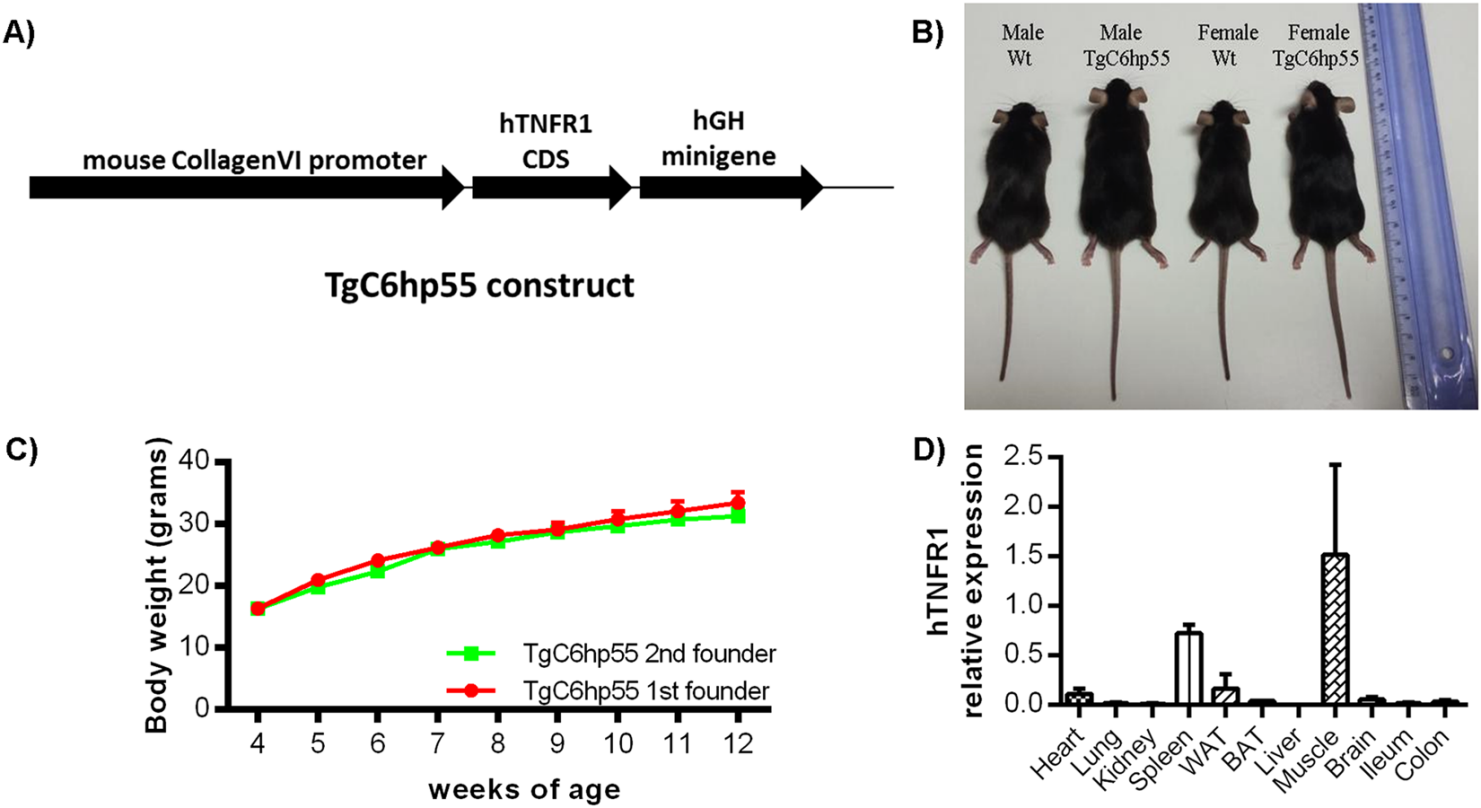
TgC6hp55 mice express the human TNFR1 under the CollagenVI promoter and show increased body size. **(A)** The construct used for the generation of the TgC6hp55 mice. **(B)** Macroscopic comparison of male and female transgenic mice versus their wild type littermates at the age of 3 months. **(C)** Comparison of the body weights of the two founders and the transgenic mice coming from them. At all time points there is no statistically significant difference between the two curves. **(D)** Q-PCR analysis for the detection of the human TNFR1 mRNA in different tissues from n = 3 TgC6hp55 mice. WAT, abdominal white adipose tissue; BAT, brown adipose tissue.

For the generation of the TgC6hp55 mice, a 1.5 kb fragment encompassing the *hTNFR1* coding DNA sequence (CDS)^26^ was inserted downstream of the 7.5 kb 5′-flanking region of the *CollagenVI*(*a1*) promoter^25^, followed by a 2.1 kb *hGH* minigene^27^ (Fig. 1A). Transgenic integration was confirmed by PCR analysis. One male mouse that was found to carry the transgene was used for the establishment of the TgC6hp55 mouse line (Founder 1) (Fig. 1B). Next, following the same procedure, we generated a second mouse carrying the same transgene (Founder 2). The mouse lines were backcrossed to a C57BL/6J background for more than 8 generations. Q-PCR analysis of the transgene copy number showed the insertion of 2 copies into the mouse genome of the first founder and of 14 copies into the mouse genome of the second founder. Despite the difference in copy number between the two founders, the two transgenic mouse lines exhibited exactly the same body weight, suggesting that the difference in copy number does not have a different effect on the gross phenotype (Fig. 1C). Therefore, in order to avoid extra complications due to the increased copy number described in the second founder, we focused our main analysis to the first founder.

Q-PCR analysis in different tissues of the TgC6hp55 mice confirmed human TNFR1 expression from the transgene. High expression levels were found in skeletal muscle and spleen, followed by lower levels of expression in abdominal white adipose tissue (WAT) and heart, expression at detection levels in lung, kidney, brown adipose tissue (BAT), brain, ileum and colon and no expression (or expression below detection levels) in the liver (Fig. 1D). Skeletal muscle, heart and intestine are typical sites of CollagenVI expression, while lung, kidney, liver, brain and adipose tissue have also been reported to express CollagenVI in some cases^23, 25^. As for the spleen, although it is not regarded as a typical CollagenVI expressing tissue, we have recently shown that there are cells in the spleen that express CollagenVI^28^. Therefore the expression pattern of the transgene is in agreement with the mesenchymal expression of CollagenVI in different tissues.

### TgC6hp55 transgenic mice show enhanced somatic growth accompanied by alterations in various parameters of their physiology

The phenotypic characterization of the TgC6hp55 mice in collaboration with the German Mouse Clinic (GMC) included macroscopic examination as well as examination regarding bone/skeletal morphology, hematological and biochemical blood parameters, immunology, neurology, nociception, allergies, cardiovascular system and eye morphology/function^29, 30^. (The phenotyping pipeline used is described in detail in the Materials and Methods section).

**Figure 2:**
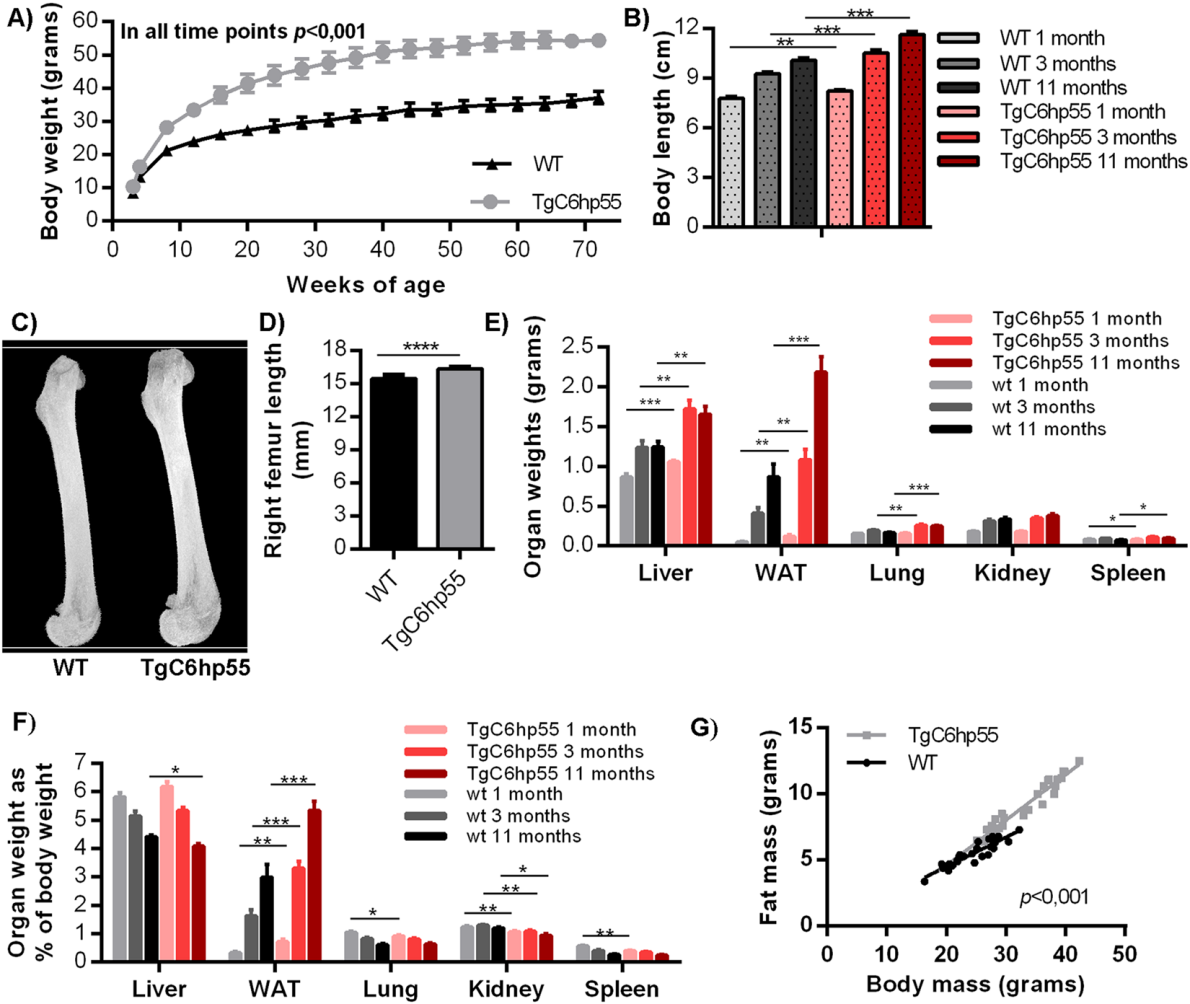
TgC6hp55 mice show enhanced somatic growth with some additional obesity-like characteristics. **(A)** Body weight curve performed in wt (n = 10) and TgC6hp55 (n = 10) mice coming from Founder 1 from the age of 3 weeks till the age of 72 weeks. **(B)** Comparison of the body length of wt and TgC6hp55 mice at the age of 1, 3 and 11 months. As body length we measured the distance from the tip of the mouse nose to the base of its tail. At 1 month n = 10 wt and n = 10 TgC6hp55 mice were compared, while at 3 months n = 8 wt and n = 8 TgC6hp55 and at 11 months n = 7 wt and n = 9 TgC6hp55. **(C)** Comparison of the right femur length of wt (n = 7) and TgC6hp55 (n = 6) mice at the age of 3 months using microCT analysis. **(D**) Quantification of the microCT analysis data presented in (**C**). **(E**) Comparison of liver, abdominal white adipose tissue (WAT), lung (both lungs), kidney (both kidneys) and spleen weight of the wt and TgC6hp55 mice shown in (**B**) at the age of 1, 3 and 11 months. **(F)** Comparison of the normalized organ weights from (**E**) expressed as % of the mouse body weight at the age of 1, 3 and 11 months. **(G)** Linear regression analysis of the fat tissue mass in correlation with body mass in wt (n = 30) and TgC6hp55 (n = 29) at the age of 13 weeks. Data represent mean ± SEM.

TgC6hp55 mice showed by 3 weeks of age an increased body weight as compared to their wild type littermates (Fig. 2A and Supplementary Fig. S1) and an increased body length as assessed in three different time points (1 month, 3 months and 11 months old) (Fig. 2B). MicroCT analysis of the right femur showed that TgC6hp55 mice also had increased bone length (Fig. 2C and D). At the same time points, as shown in Fig. 2E, most of the organs from the transgenic mice were heavier than their controls’. Furthermore, as shown in Fig. 2F, the normalized weights of these organs did not differ significantly from their controls’, indicating a generally enhanced somatic growth. Only white adipose tissue’s (WAT) normalized weight was increased in the transgenic mice hinting towards an additional obesity-like phenotype. A whole body composition analysis at the age of 13 weeks (also repeated at 19 weeks) using quantitative Nuclear Magnetic Resonance (qNMR) showed an increase in both lean and fat mass in the transgenic mice (Supplementary Fig. S2). When normalized according to total body weight, the fat mass was found to be increased in the TgC6hp55 mice while the lean mass slightly decreased (Supplementary Fig. S2). Analysis using linear regression analysis of the fat tissue mass weight in correlation to body weight (Fig. 2G) according to Packard and Boardman^31^ confirmed that the normalized total fat tissue weight in the TgC6hp55 mice is bigger than in wt.

**Figure 3:**
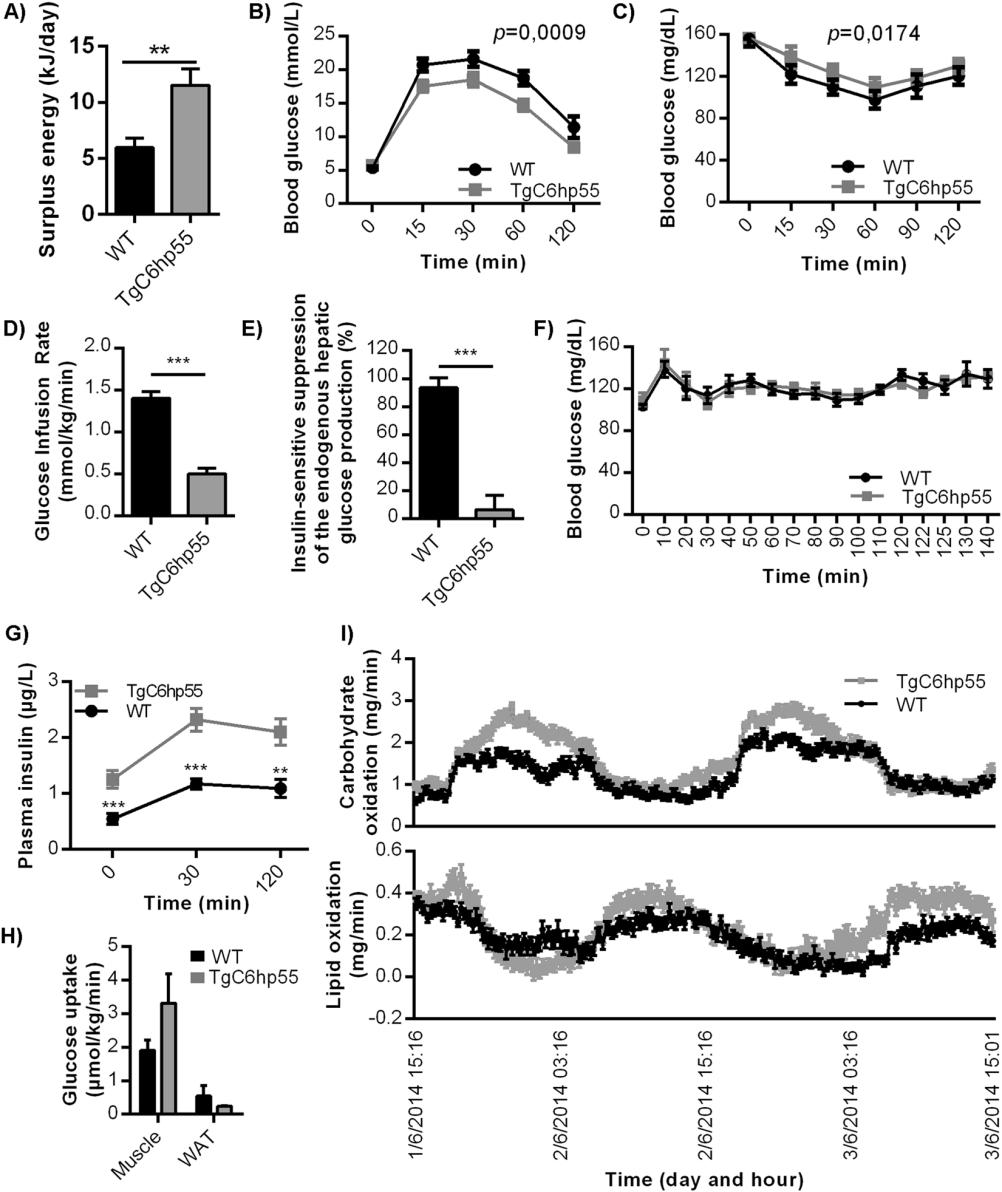
TgC6hp55 mice show increased energy surplus and are normoglycemic despite perturbed insulin sensitivity. **(A)** Comparison of the daily energy surplus between wt and TgC6hp55 mice (n = 15 wt and 11 TgC6hp55). **(B)** Intraperitoneal Glucose Tolerance Test (ipGTT) performed in wt (n = 16) and TgC6hp55 (n = 13) mice. *p* value represents the comparison between the Area Under the Curve for the wt versus TgC6hp55 mice. **(C)** Intraperitoneal Insulin Tolerance Test (ipITT) performed in wt (n = 16) and TgC6hp55 (n = 13) mice. *p* value represents the comparison between the Area Under the Curve for the wt versus TgC6hp55 mice. **(D)** Comparison of the Glucose Infusion Rate needed in order to retain euglycemia in the mice during the hyperinsulinemic-euglycemic clamp (n = 7 wt and 6 TgC6hp55 mice). **(E)** Comparison of the Insulin-Sensitive Suppression of the Endogenous Hepatic Glucose Production during the hyperinsulinemic-euglycemic clamp in n = 8 wt and n = 7 TgC6hp55 mice. **(F)** Blood glucose levels during the basal period of the hyperinsulinemic-euglycemic clamp. **(G)** Measurement of the Glucose Stimulated Insulin Secretion during the ipGTT shown in Fig. 3C. (**H**) Comparison of the glucose uptake from the skeletal muscle (gastrocnemius muscle) and from the white adipose tissue (epididymal white adipose tissue) in wt (n = 6) and TgC6hp55 (n = 6) mice. **(I)** Comparison of the carbohydrate and lipid oxidation during a 2-days calorimetry in n = 16 wt and n = 15 TgC6hp55 mice. Only the differences observed in the carbohydrate oxidation were statistically significant.

**Figure 4:**
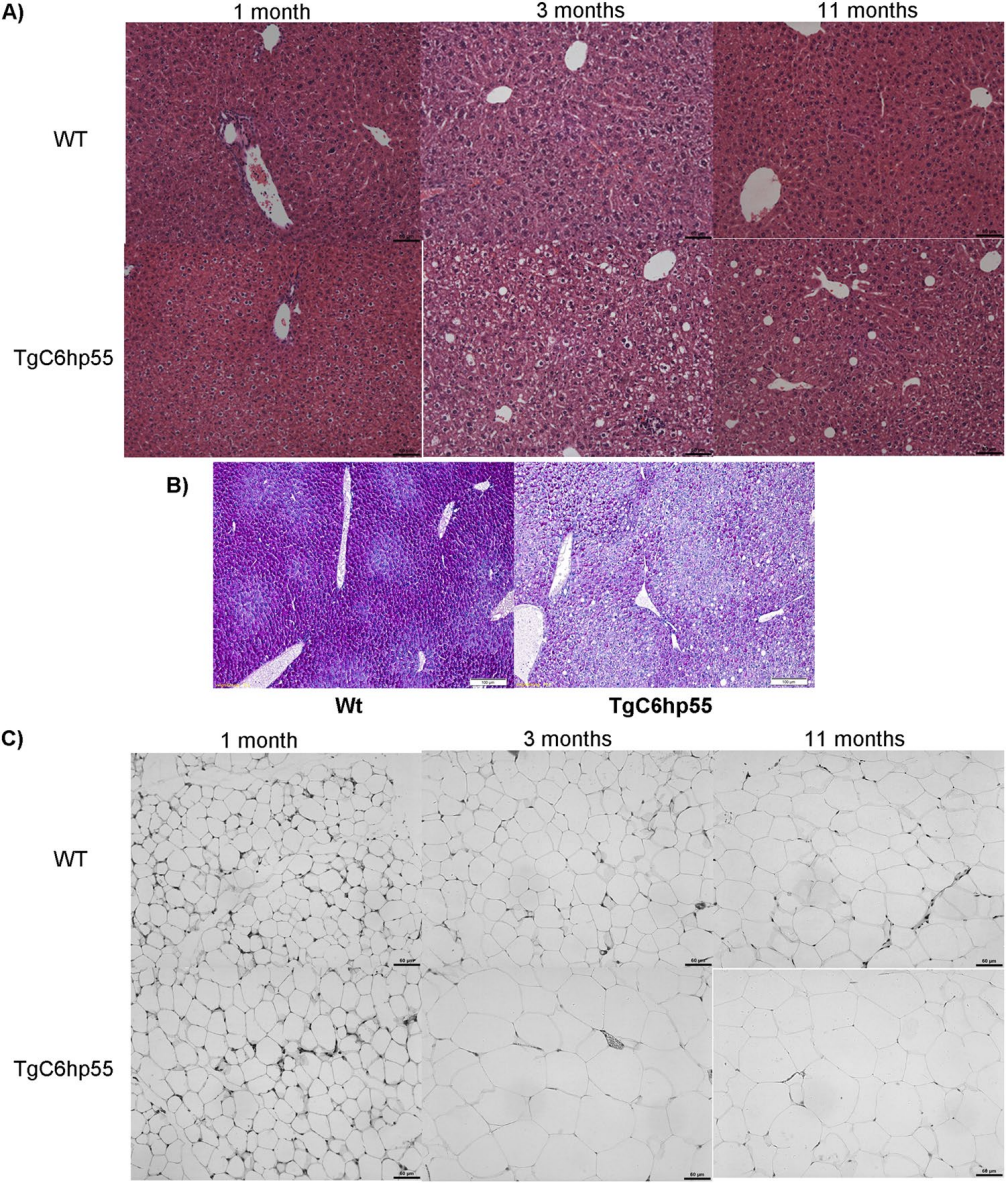
TgC6hp55 mice show histopathological alterations in their liver and white adipose tissue. **(A**) Histopathological assessment of the progressive lipid accumulation in the livers of wt and TgC6hp55 mice at the age of 1, 3 and 11 months (H/E staining). **(B)** Histopathological comparison of the glycogen content of the livers of wt and TgC6hp55 mice at the age of 3 months (PAS staining). **(C)** Histopathological comparison of the adipocyte size from the abdominal white adipose tissue of wt and TgC6hp55 mice at the age of 1, 3 and 11 months (H/E staining). N = 7–10 wt and n = 7–10 TgC6hp55. (**A**) and (**C**) are in 200x magnification/scale = 60 μm while (**B**) in 100x magnification/scale = 100 μm. PAS, Periodic acid-Schiff Stain.

A 2-days indirect calorimetry analysis including monitoring of food uptake revealed that TgC6hp55 mice are both hyperphagic (increased energy uptake) and hypermetabolic (increased energy expenditure) (Supplementary Fig. S3) with their total daily energy surplus (daily energy uptake of metabolizable energy minus daily energy expenditure) being positive and increased (Fig. 3A). Additionally, examination of the glucose homeostasis and insulin sensitivity using an intraperitoneal glucose tolerance test (ipGTT) (Fig. 3B), an intraperitoneal insulin tolerance test (ipITT) (Fig. 3C) and a hyperinsulinemic-euglycemic clamp [including glucose infusion rate (GINF) (Fig. 3D) and insulin sensitive suppression of endogenous hepatic glucose production (Fig. 3E)] revealed that the TgC6hp55 mice were insulin resistant. Nevertheless, they stayed normoglycemic under steady-state conditions (Fig. 3F) due to the hyperinsulinemia they were exhibiting accompanied by an enhanced glucose stimulated insulin secretion (GSIS) (Fig. 3G) which both resulted in normal glucose uptake from the skeletal muscle and the WAT (Fig. 3H). This enhanced glucose-stimulated insulin secretion can also explain the decreased blood glucose levels observed in the TgC6hp55 mice during the ipGTT shown in Fig. 3B. Finally, the 2-days indirect calorimetry uncovered an interesting increase in carbohydrate utilization by the transgenic mice during the night (the time when nocturnal animals like mice have increased energy demands), while lipid oxidation was unaffected (Fig. 3I). In agreement to the above described metabolic characteristics of the TgC6hp55 mice, histological analysis detected a progressive lipid accumulation in the liver of the TgC6hp55 mice which developed into a moderate macrovesicular hepatic steatosis (Fig. 4A) accompanied by decreased hepatic glycogen storage (Fig. 4B) as well as increased white adipocyte size (Fig. 4C). Furthermore and in correlation with these findings, gene expression analyses performed in the liver of the TgC6hp55 mice revealed the regulation of genes functionally associated with metabolism of lipids, carbohydrates and proteins, metabolic diseases like obesity and inflammatory processes (GSE95345).

Finally, additional differences associated with the above-described phenotype were found in bone morphology and metabolism (major differences) as well as in hematological, biochemical and immunological exams (minor differences) and are depicted in Supplementary Figs S4 and S5.

### The phenotype of the TgC6hp55 mice is TNF-, TNFR1- and transgene insertion-independent

To examine how the incorporation of the CollagenVI promoter-driven hTNFRI transgene in the genome led to the above-described phenotype first we aimed to verify that this phenotype depends upon TNF/TNFR1 signaling. For this purpose we crossed TgC6hp55 mice with mice in which either TNF (*Tnf* ^−/−^ mice)^32^ or TRADD – TNF Receptor Associated Death Domain - (*Tradd* ^*D/D*^ mice)^33^ have been genetically deleted. In the *Tnf* ^−/−^ mice, TNF is not produced and therefore it does not activate TNFR1, since TNF is the main ligand that binds to and activates TNFR1 (which however can also be activated by Lymphotoxin a)^34^. Additionally, in the *Tradd* ^*D/D*^ mice, TRADD which is a major component of most of the signaling cascades downstream TNFR1 (including apoptosis and NFkB activation)^33, 35^ is missing, so after the activation of (mouse or human) TNFR1, these downstream signaling cascades cannot be activated.

**Figure 5:**
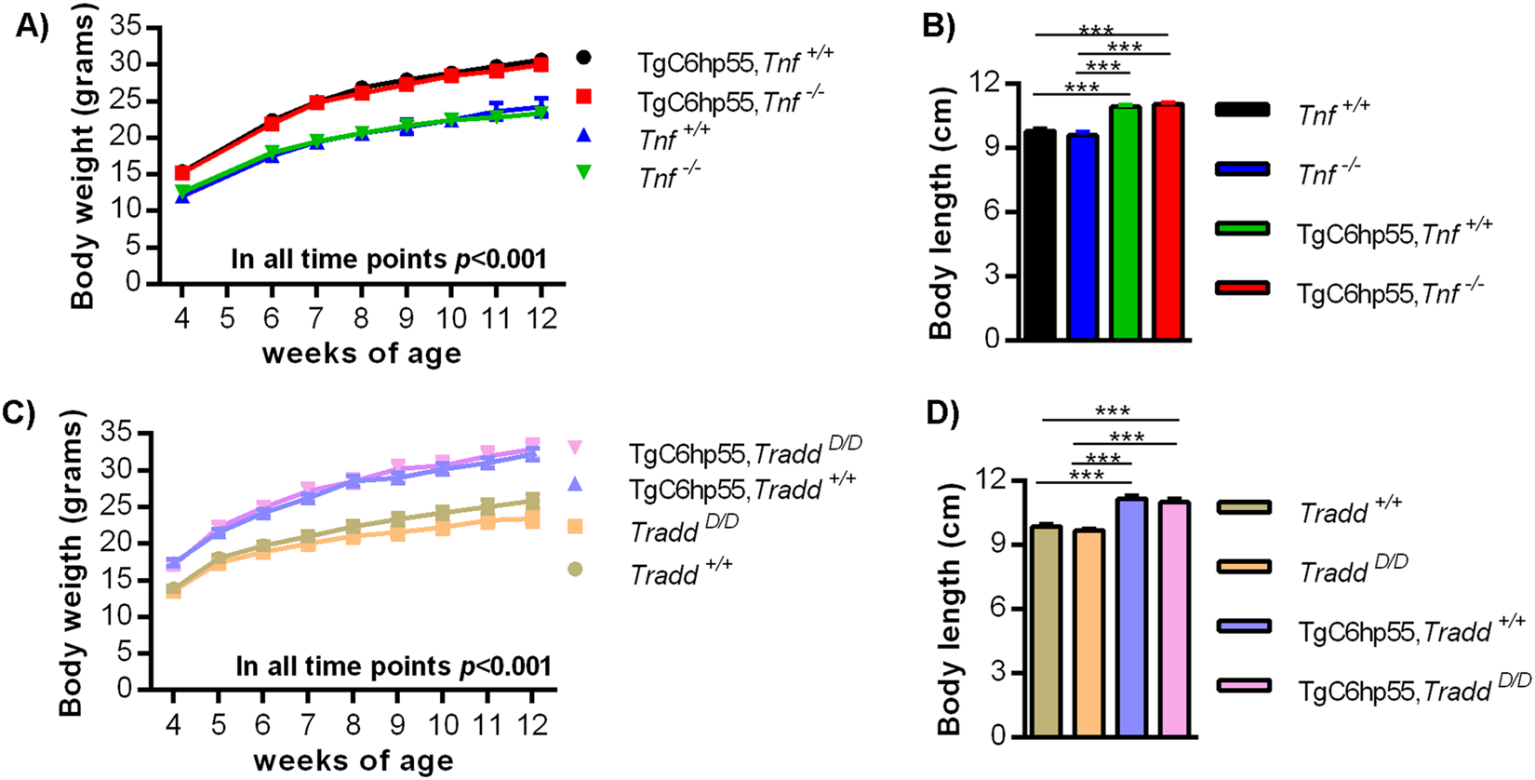
The TgC6hp55 phenotype is primary and independent of TNF, TNFR1 and insertion effects. **(A)** Body weight curve of *Tnf* ^+/+^ (n = 12), *Tnf* ^−/−^(n = 24), TgC6hp55*Tnf* ^+/+^ (n = 12) and TgC6hp55*Tnf* ^−/−^ (n = 21) mice. **(B**) Body length comparison of *Tnf* ^+/+^ (n = 6), *Tnf* ^−/−^(n = 11), TgC6hp55*Tnf* ^+/+^ (n = 6) and TgC6hp55*Tnf* ^−/−^ (n = 9) mice. **(C)** Body weight curve of *Tradd* ^+/+^ (n = 15), *Tradd* ^*D/D*^ (n = 11), TgC6hp55*Tradd* ^+/+^ (n = 19) and TgC6hp55*Tradd* ^*D/D*^ (n = 8) mice. **(D)** Body length comparison of *Tradd* ^+/+^ (n = 8), *Tradd* ^*D/D*^ (n = 7), TgC6hp55*Tradd* ^+/+^ (n = 7) and TgC6hp55*Tradd* ^*D/D*^ (n = 7) mice. Data represent mean ± SEM.

To our surprise, both TgC6hp55*Tnf* ^−/−^ mice and TgC6hp55*Tradd* ^*D/D*^ mice didn’t show any amelioration of the phenotype. They had increased body weight (Fig. 5A,C) and body length (Fig. 5B,D) as well as increased organ weights. Additionally, their normalized, as a % of body weight, organ weights didn’t show any major differences apart from normalized WAT weight that was still increased (Supplementary Fig. S6). Finally, in the histological examination, they displayed lipid infiltrations in their liver and increased adipocyte size in their WAT (Supplementary Fig. S7). All these results suggest that the TgC6hp55 phenotype is TNF- and Tnfr1 signaling**-**independent.

Another scenario we had to take into consideration was that of a transgene insertion locus effect. Using the Targeted Locus Amplification technique (TLA)^36^, we managed to identify the single insertion site of the transgene to be located in Chromosome 10 and specifically in the area Chr10:77533719-77533707. Additionally, the insertion was found to interrupt the genes of Icoslg and Dnmt3l that lie adjacent to this region by deleting some of their exons and therefore, in order to verify that the insertion of the transgene in the specific position and the local disturbances it caused to the native DNA were not the cause of the TgC6hp55 phenotype, we generated a second founder (as described previously). This founder also exhibited the same phenotype of increased body size as the first founder (Supplementary Fig. S8), confirming that the TgC6hp55 phenotype is not secondary to an insertion effect.

### The *hGH* minigene is expressed in the TgC6hp55 mice leading to local and systemic hGH production and downregulation of endogenous GH

**Figure 6:**
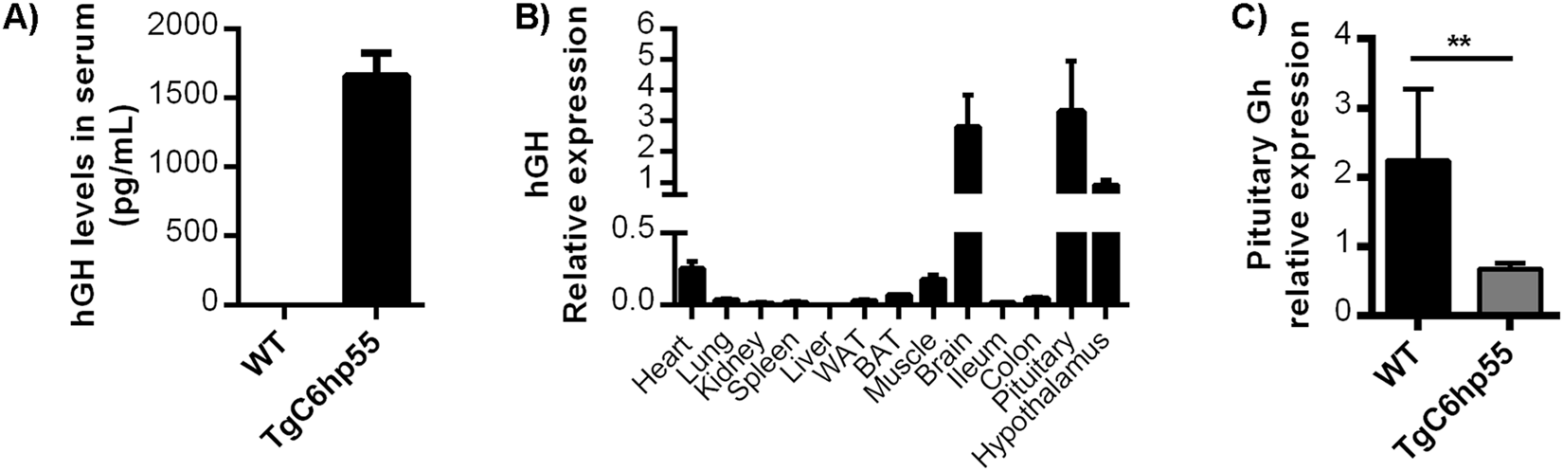
Detection of the *hGH* minigene expression in the TgC6hp55 mice. **(A)** Detection of the human growth hormone in the serum of TgC6hp55 mice using ELISA (n = 10 wt and n = 10 TgC6hp55 mice). **(B)** Q-PCR analysis for the detection of the *hGH* mRNA in different tissues of 3 TgC6hp55 mice. WAT, abdominal white adipose tissue; BAT, brown adipose tissue. **(C)** Q-PCR comparison of the mouse growth hormone produced by the pituitary of wt (n = 6) and TgC6hp55 (n = 6) mice at the age of 2, 5 months. Data represent mean ± SEM.

Since there seemed to be no other possible explanation for the TgCh6p55 phenotype, we decided to investigate whether the *hGH* minigene was expressed. Indeed the hGH protein was detected in the serum of TgC6hp55 mice at levels of around 1.6 ng/mL as assessed by ELISA (Fig. 6A). Analysis with qPCR in different tissues detected high levels of the *hGH* mRNA in the brain (both in the whole brain and in the pituitary and the hypothalamus) and also expression in the heart and the skeletal muscle. The rest of the tissues tested (lung, kidney, spleen, WAT, BAT, ileum and colon) showed very low expression levels of hGH while no expression was detected in the liver (Fig. 6B). As explained before, the skeletal muscle and the heart are two tissues were the CollagenVI promoter is expected to be active while its expression, especially the ectopic expression in the brain, has also been described in the rest of the tissues^23, 25^. These results indicate that the expression of our *hGH* minigene is controlled by the CollagenVI promoter. The fact that the expression pattern of the *hGH* minigene is not identical with that of the TNFR1 could be explained by the potentially differential regulation of the hTNFR1 and hGH RNAs in the different tissues. For example while hTNFR1 RNA is known to be minimally regulated at the mRNA stability level, evidence suggests that hGH mRNA follows more complex patterns of regulation e.g. in brain tissue^27^.

Finally, as previously reported in other transgenic mouse lines^15, 16, 18^, the expression of the hGH in the brain and especially in the pituitary and the hypothalamus of the TgC6hp55 mice led to an almost 50% downregulation of the endogenous *mGh* expression in the pituitary. (Fig. 6C).

### Pharmacological inhibition of the GH signaling reverses most of the TgC6hp55 phenotypic characteristics

**Figure 7:**
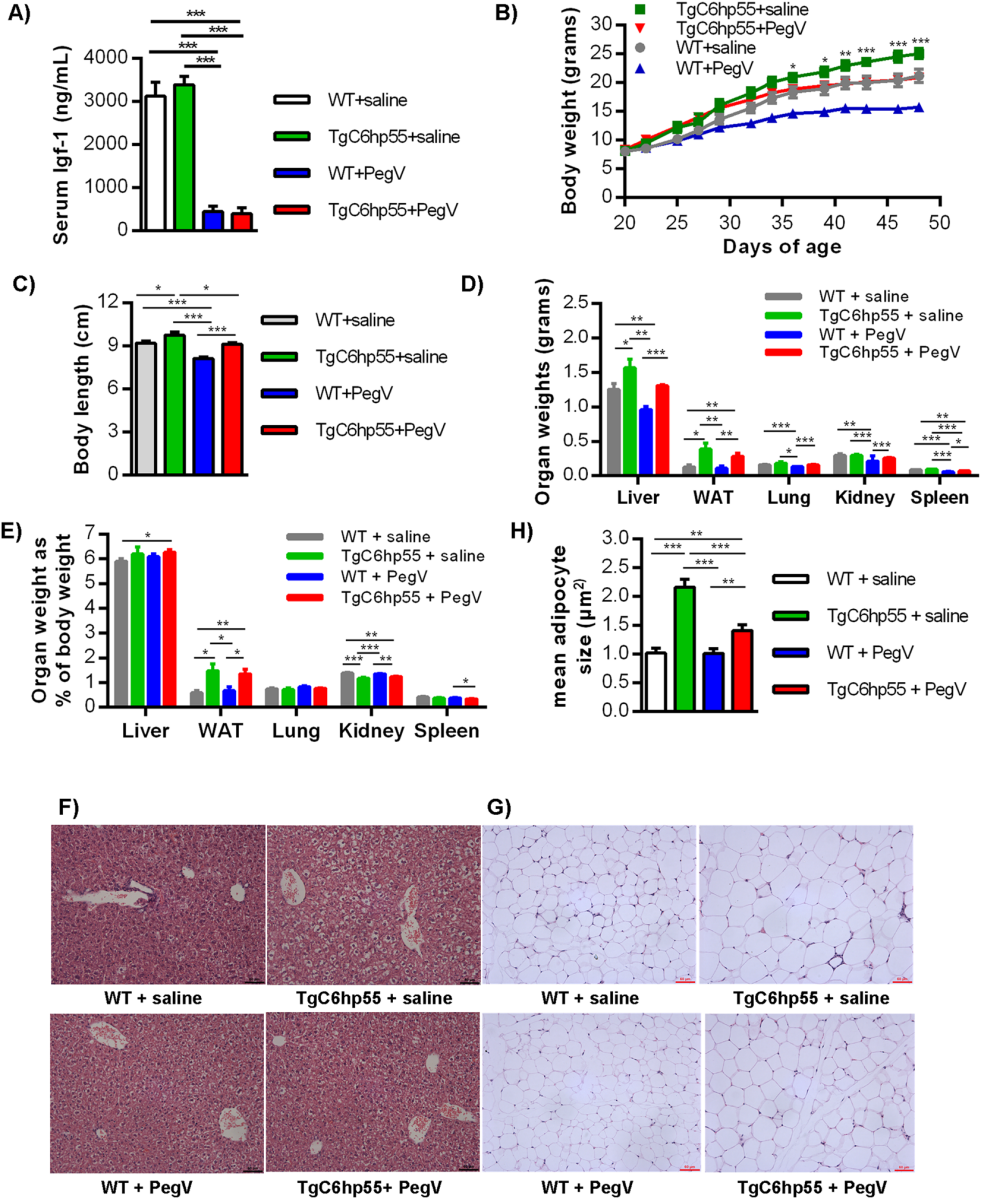
Pharmacological inhibition of the GH signaling reverses most of the TgC6hp55 phenotypic characteristics. 4 groups of mice: 8 wt received saline, 8 wt received pegvisomant (PegV), 8 TgC6hp55 received saline and 8 TgC6hp55 received PegV. **(A)** Serum levels of Igf-1 in all 4 groups. **(B)** Body weight curve. *Represent comparison between TgC6hp55+saline versus TgC6hp55+PegV. **(C)** Body length comparison. **(D)** Comparison of liver, abdominal white adipose tissue (WAT), lung (both lungs), kidney (both kidneys) and spleen weight among 4 groups. **(E)** Comparison of the normalized organ weights from (7D) expressed as % of the mouse body weight. **(F)** Histopathological assessment of the lipid accumulation in the livers of all 4 groups (H/E staining). **(G)** Histopathological comparison of the adipocyte size from the abdominal white adipose tissue of all 4 groups (H/E staining). **(H)** Quantification of the adipocyte size shown in Fig. 7F. (**F**) and (**G**) are in 200x magnification/scale = 60 μm. Data represent mean ± SEM.

To examine whether hGH expression is causally involved in the development of the TgC6hp55 phenotype, we used a growth hormone receptor antagonist (pegvisomant or PegV), an antibody which pharmacologically inhibits the growth hormone signaling by binding to the GH receptor and blocking the binding of the GH^37^. Initially, we separated the mice into 4 different groups: wt mice treated with PegV (wt+PegV), wt mice treated with saline (wt+saline), TgC6hp55 mice treated with PegV (TgC6hp55+PegV) and TgC6hp55 mice treated with saline (TgC6hp55+saline). Starting at 20 days of age (just before the TgC6hp55 mice begin to show a difference in body weight), the mice were injected 3 times per week following a preventive administration scheme. In order to validate the effectiveness of Pegvisomant in blocking GH signaling we measured the levels of serum IGF-1^38–40^ in all 4 groups. As shown in Fig. 7A, both groups treated with PegV showed a decrease in the circulating levels of IGF-1, confirming its effectiveness. After the 4-weeks treatment, the TgC6hp55+PegV mice were similar to the wt+saline mice in terms of body weight and body length and were significantly smaller than the TgC6hp55+saline mice (Fig. 7B,C). As for the raw and normalized organ weights (Fig. 7D,E), the results were not conclusive, since some organs (like liver or lungs) did not grow as much after the PegV treatment as compared to the saline treatment, while others (like WAT) did grow more. Probably at that early age at which we examined the mice the differences are yet not so big and also the treatment might need a longer duration in order to affect organ size substantially. However we cannot exclude the small probability that the effectiveness of PegV and/or some hGH-independent parts of the TgC6hp55 phenotype could be attributed to other mechanisms like the activation of the prolactin receptor or the expression and action of the TNFR1 transgene or some complex insertion effects. Finally, GH inhibition in the TgC6hp55+PegV mice prevented the increased hepatocyte lipid storage in their livers which appeared completely normal (Fig. 7F). In the WAT, the adipocytes of these mice, despite being bigger than the ones of the wt+saline group, they were significantly smaller than those of the TgC6hp55+saline group (Fig. 7G,H). Collectively all these data confirm that the development of the main phenotypic characteristics of the TgC6hp55 mice are prevented by the blockade of growth hormone signaling, indicating that the hGH is a key player in the development of the TgC6hp55 phenotype.

Furthermore, it is well known that GH can act both locally in an autocrine/paracrine way and systemically either directly or indirectly through IGF-1^41, 42^. Therefore we used the circulating IGF-1 levels as an indicator of the possible systemic effects of the hGH^38^. As shown again in Fig. 7A, the transgenic mice had the same levels of serum IGF-1 as their wt controls. This finding means that the circulating hGH is effective either independently of the liver-produced IGF-1 or through local production and action in different tissues or both.

## Discussion

Although this is not the first time that the *hGH* minigene has been found to be expressed in a transgenic mouse line^12–18^, in this work we provide original evidence for the effects of this expression when it is driven by the mesenchymal CollagenVI promoter. As shown in Table 1, up to date, the “unexpected” expression of the *hGH* minigene and its consequences on the mouse phenotype have been studied and described only in a few mouse lines which used promoters that are specific for either the pancreas or the liver or the nervous system and therefore led to a more or less confined expression of the hGH in specific areas like the brain or the pancreatic islets. On the contrary, in our transgenic mouse line, the TgC6hp55, the *hGH* minigene expression was found in a series of tissues under the control of the CollagenVI promoter and it is the first time to our knowledge, that the expression of a *hGH* minigene is reported to drive enhanced somatic growth as well as a plethora of alterations in various parameters of the mouse physiology as described before. Additionally, by the pharmacological blockade of GH signaling we present direct evidence of the causal relationship between the *hGH* minigene expression and the observed phenotype.

**Table 1:** Other publications studying the effects of hGH minigene expression.

Publication	Mouse line	Specific for	Site of expression	Serum hGH	Detection method	Effects attributed to the hGH
Pruniau *et al*.^18^	AlfpCre	Hepatocytes	Hypothalamus, pituitary	Not examined	Q-PCR, ELISA	liver steatosis, reduced growth
Nuytens *et al*.^16^	Nbea^+/−^GH240B	no promoter	Hypothalamus, pituitary	No	Q-PCR, WB, ELISA, IF	reduced growth, obesity,
Browers *et al*.^12^	Pdx1Cre^Late^	pancreatic α- and β-cells	pancreatic β-cells	Not examined	Q-PCR, WB, IHC	altered β-cell physiology and glucose/insulin regulation
Browers *et al*.^12^	MIPCre, RIPCre	pancreatic β-cells	pancreatic β-cells	Not examined	Q-PCR, WB, IF	
Baan *et al*.^13^	MIPFoxM1	pancreatic β-cells	pancreatic β-cells	No	Q-PCR, WB, ELISA	altered β-cell physiology
Declercq *et al*.^15^	NestinCre	nervous system	Hypothalamus, pituitary	Not examined	Q-PCR, ELISA	hypopituitarism, reduced growth, liver steatosis, behavioral problems
Oropeza *et al*.^17^, Carboneau *et al*. 2016	MIPCreERT^1L*phi*^	Pancreatic β-cells	pancreatic β-cells, hypothalamus	Not examined	Q-PCR, WB	altered β-cell physiology and glucose/insulin regulation

WB, Western Blot; IHC, Immunohistochemistry; IF, Immunofluorescence.

In some of the studies referred in Table 1, local expression of the *hGH* minigene in the mouse pituitary and hypothalamus led to decreased somatic growth due to downregulation of the endogenous mouse Gh^15, 16, 18^, while in the rest, where the minigene was not expressed in the brain, no alterations in body size were found^12, 13, 17^. An important issue not clarified in most of these studies^12, 14, 15, 17, 18^ is whether the locally produced hGH makes its way into the circulation or not. The 2 studies that looked into this issue did not detect any hGH in the circulation^13, 16^.

Interestingly though, the TgC6hp55 mice exhibited increased body size with unchanged systemic Igf-1 levels, in spite of an almost 50% downregulation of the endogenous Gh. This phenotype could be attributed either to the local or to the systemic effects of the hGH or both. On the one hand, it has been shown that GH can act both locally and systemically without affecting the serum Igf-1 levels^38, 40^. On the other hand, locally produced GH, even if it accesses the circulation, may still exhibit no systemic effects^38^. Most of the tissues examined in the TgC6hp55 mice scored positive for hGH expression, which suggests local activity. Additionally, the circulating levels of hGH appear not high enough to increase systemic Igf-1 levels, but they still seem to drive systemic effects as for example in the liver where hGH is not locally produced. Therefore, the most probable explanation in the TgC6hp55 mice is that hGH acts mainly locally with some extra effects being attributed to its systemic action. Of course all these hypotheses would be interesting to be further addressed in the future.

Another interesting characteristic of the TgC6hp55 mice which we studied and described thoroughly is the perturbed insulin sensitivity and carbohydrate metabolism. Transgenic mice expressing the *hGH* minigene have also exhibited analogous effects^12–14, 17^. However, these mice were expressing the *hGH* minigene in the pancreatic islets or specifically in β-cells, making it much easier for the hGH to directly interact in an autocrine/paracrine way with the insulin production. Moreover, it is known that hGH can bind to and activate also the prolactin receptor^19^ and therefore mimic the effects of lactogens on mouse β-cells^43^. In correlation with previous studies^12^, we also found increased serotonin expression in the pancreatic islets of the TgC6hp55 mice (Supplementary Fig. S9). Since serotonin is used as a marker of prolactin receptor stimulation in the pancreas^44, 45^, this finding suggests that at least in the pancreas of the TgC6hp55 mice hGH binds to and activates the prolactin receptor. Therefore some of the effects described regarding the carbohydrate metabolism could be explained by this mechanism^12^. Moreover, the activation of the prolactin receptor by the hGH could also explain some other phenotypic characteristics of the TgC6hp55 mice such as hepatomegaly^14, 26, 28^, while it is contradictory to other findings in these mice like the increased bone mineral density and the increased adipose tissue mass^30, 46^. However, the fact that the pharmacological blockade of the growth hormone receptor reversed most of the main phenotypic characteristics of the TgC6hp55 mice (enhanced somatic growth, hepatic steatosis, increased adipocyte size etc.), suggests that the major actions of the hGH in mediating the TgC6hp55 phenotype are through this receptor, while the activation of the prolactin receptor plays a more secondary role. Nevertheless, it would be interesting in future studies to address this question in more detail.

Furthermore, the TgC6hp55 mice also exhibited an interesting phenotype of increased food intake in conjunction with increased metabolic rate and a preference in mobilizing carbohydrates in order to cover their increased energy demands during night, that has not been previously described in correlation with the *hGH* minigene.

The *hGH* minigene-expressing transgenic mice with a reduced endogenous Gh production and a reduction in body growth described above^15, 16, 18^ also show perturbations in lipid metabolism (increased triglycerides, increased adipose tissue mass, liver steatosis and deregulated genes that take part in lipid metabolism) similar to our findings, despite the increased body size of the TgC6hp55 mice^47^. However, the same features have been previously described in the context of insulin resistance and/or obesity^48, 49^, like in the transgenic mice overexpressing bovine GH in their central nervous system causing hyperphagia-induced obesity^50^. This mechanism of hyperphagia-induced obesity could explain the findings of increased adipose tissue mass in the TgC6hp55 mice and it could present a new secondary ‘side-effect’ of the *hGH* minigene expression in the brain. Of course we cannot exclude an additional effect of the locally (even in very small amounts) produced hGH on WAT, since the existing literature regarding such an issue is still debating^51–53^. Finally, the rest of the alterations observed in the skeletal, biochemical and hematological examination of the TgC6hp55 mice have also never been described before in conjunction with the hGH minigene expression.

Altogether the results from our analysis show that the unplanned expression of the hGH minigene, if/when it happens and depending on the context in which it acts, can have a plethora of effects that are or are not always restricted to the specific tissue of expression. More specifically, we have shown that the local expression of this minigene in the CollagenVI expressing mesenchymal cells can affect various aspects of the mouse physiology not only locally but also systemically. However, we cannot safely distinguish which of the phenotypic characteristics described in the TgC6hp55 mice can primary be attributed to the hGH minigene expression and which are secondary to the generally enhanced growth. The results of our study along with other recent studies underline that it is crucial for all using the hGH minigene in our mouse models to examine if it is expressed or not and the possible effects it might have to the described phenotypes, as the lack of visible growth changes does not necessarily mean that the minigene is not expressed. Finally, it would be important to examine these mice more systemically and to not only focus locally because, as we have shown in the present work, the ‘side-effects’ of the hGH minigene expression might not always be confined to a local level.

## Materials and Methods

### Mice

All mice were bred and maintained on a C57BL/6J genetic background in the animal facilities of the Biomedical Sciences Research Center “Alexander Fleming” under specific pathogen-free conditions. Experiments were approved by the Institutional Committee of Protocol Evaluation in conjunction with the Veterinary Service Management of the Hellenic Republic Prefecture of Attika according to all current European and national legislation and were performed in accordance with relevant guidelines and regulations. All mice were housed in standard cages (wood-shaving bedding) on a 12-hour day/night cycle and were fed a standard rodent chow.

### Generation of TgC6hp55 transgenic mice

The procedure for the generation of the TgC6hp55 mice has been described in the Results section. Pronuclear injection of the transgene into fertilized (C57BL/6JXCBA/J)F2 oocytes was performed in the BSRC Alexander Fleming Transgenesis Facility. The CollagenVI(a1) promoter was kindly provided by G. Bressan (University of Padova, Padova, Italy)^25^. The hTNFR1 CDS was kindly provided by D. Wallach (The Weizmann Institute of Science, Rehovot, Israel)^26^. The hGH minigene was kindly provided by R. Perlmutter (Howard Hughes Medical Institute, University of Washington, Washington, USA)^27^. For the genotyping of the mice, the primers that we used were: Forward: AGGAAATGGGTCAGGTGGAG and Reverse: CTCAATCTGGGGTAGGCACA. The conditions of the PCR were as following: annealing for 30 seconds at 66 °C declining by 1 °C per cycle until 57 °C and then at 57 °C for 30 more cycles; elongation for 1 minute at 72 °C. The generation of the *Tnf* ^−/−^ mice has been described previously^32^ while the *Tradd* ^*D/D*^ mice were kindly provided to us by M. Pasparakis (University of Cologne, Cologne, Germany)^33^.

In all the experiments we tried to use equal numbers of males and females from each genotype. Additionally, in all experiments the mice that were used were littermates or at least cohoused since their weaning day.

### Q-PCR

All reactions were performed in 96 well plates in a PTC200 Thermal Cycler from MJ Research (USA) and the data were analyzed using Opticon Monitor 3.

### Copy number calculation

For the calculation of the number of copies that were inserted into the genome of each founder and his ancestors we isolated genomic tail DNA using phenol:chlorophorm extraction. All the samples were diluted to a final concentration of 12, 5 ng/μl and 2 μl from each sample were used in each reaction. For the multiplication of the transgene we used primers that detect the exon 3 of the hTNFR1 while MAP3K8 was used as a reference gene for normalization. Finally, the human *p55* knock-in mouse was used as a control that contains 2 copies of the human p55^54^. The primer sequences were: hTNFR1 exon3F: 5′-TAC AAT GAC TGT CCA GGC CCG-3′, hTNFR1 exon3R: 5′-GCA TTT GGA GCA GCT GAG GC-3′, MAP3K8F: 5-TCC AGG CCT GTT TCC GGC-3′, MAP3K8R: 5′-CCT CCC TCG CCG GCT TCC-3′. The Q-PCR conditions were: annealing at 59 °C for 30 sec, elongation at 72 °C for 30 sec. The reactions were performed using the Quantitect Real-time PCR kit from Qiagen (Cato No. 204143).

### hTNFR1, hGH and mGh expression levels

After the mice were sacrificed, the tissues of interest (heart, lung, kidney, spleen, liver, muscle, brain, ileum, colon, abdominal white adipose tissue, interscapular brown adipose tissue, testis, pituitary and hypothalamus) were snap-frozen in liquid nitrogen. The total RNA was isolated and DNAse treated using the Absolutely RNA Miniprep Kit from Agilent Technologies (Cat. No. 400800). Reverse transcription was performed using the M-MLV Reverse Transcriptase from Promega (Cat. No. M1705). Samples were normalized to B2M (B2M F: 5′-TTC TGG TGC TTG TCT CAC TGA-3′; B2M R: 5′-CAG TAT GTT CGG CTT CCC ATT C-3′). The primers for hTNFR1 were purchased from Qiagen (QT00216993). The primers for hGH have been described by Browers *et al*.^12^, the primers for mGh have been described by Martari *et al*.^55^. All reactions were performed using the Quantitect Real-time PCR kit from Qiagen (Cat. No. 204143). The reaction conditions were: hTNFR1: annealing at 55 °C for 30 sec and elongation at 72 °C for 30 sec; hGH: annealing at 58 °C for 30 sec and elongation at 72 °C for 40 sec.

### Phenotypic characterization

#### Body weight curve

For the body weight curves the mice were weighed once per week starting at 4 weeks. Additionally, regarding the TgC6hp55 versus their wt siblings, we also compared their body weights at 1 week, 11 days, 2 weeks and 3 weeks of age.

#### microCT

The scanning was performed in a Skyscan 1172 microCT (Brucker). The bone specimens (right femur) were wrapped with gauze soaked in PBS and stored in a freezer at −20 °C. Before the scanning, they were left to defrost at RT. The scanning parameters were set at 49 kV and 100uA, the resolution was 1332 × 960 and the filter used was Al 0,5mm. The image reconstitution was performed using the NRecon program (SkyScan, Brucker).

### German Mouse Clinic phenotypic pipeline

#### ipGTT

Mice were used for the glucose tolerance test after a 16–18 hours-lasting overnight food-withdrawal. In the beginning of the test, the body weight of mice was determined. For the determination of the fasting blood glucose level, the tip of the tail was scored using a sterilized scalpel blade and a small drop of blood was analyzed with the Accu-Chek Aviva glucose analyzer (Roche/Mannheim). Thereafter mice were injected intraperitoneally with 2 g of glucose/kg body weight using a 20% glucose solution, a 25-gauge needle and a 1-ml syringe. 15, 30, 60 and 120 minutes after glucose injection, additional blood samples (one drop each) were collected and used to determine blood glucose levels and insulin levels (30, 60 min) as described before. Repeated bleeding was induced by removing the clot from the first incision and massaging the tail of the mouse. After the experiment was finished, mice were placed in a cage with plentiful supply of water and food^56^.

Insulin levels were determined by ELISA (Mercodia, Sweden).

### Insulin tolerance test

The insulin tolerance test was conducted in the early afternoon after 6 hours food deprivation that began in the early morning. Insulin (0.7 U per kg body mass) was injected intraperitoneally after measurement of baseline blood glucose from blood samples taken from the tail vein. Further blood samples were taken 15, 30, 60, 90 and 120 minutes after injection. Glucose levels were determined using a handheld glucometer (ACCU CHECK Aviva, Roche Diagnostics/Mannheim, Germany).

### Hyperinsulinemic-euglycemic clamp

As a preparatory step, a permanent jugular vein catheter was implanted in anesthetized experimental mice 6–7 days before the clamp study. For the hyperinsulinemic-euglycemic clamp, mice were food deprived for 6 hours beginning in the early morning. Mice were unrestrained and conscious during the procedure but placed in oversized rat restrainers and warmed by warming pads. To administer solutions catheters were connected to syringes in CMA402 pumps (Axel Semrau, Sprockhoevel, Germany). After 110 minutes of primed-continuous [3-^3^H] glucose infusion (1.85 kBq/min), a blood sample was collected to determine plasma insulin, glucose and [3-^3^H] glucose concentrations to calculate basal endogenous glucose production. Thereafter, a [3-^3^H] glucose infusion (3.7 kBq/min) containing insulin (15 pmol·kg^−1^·min^−1^; HumulinR, Lilly, Indianapolis, IN) begun. Blood glucose concentrations were monitored in intervals of 10 minutes. Target glycemia was achieved by adjusting glucose infusion rates. 120 minutes after beginning of the experiment, 2-[^14^C] deoxyglucose (370 kBq) was injected intravenously to assess rates of tissue specific glucose uptake. After conclusion of the experiment, mice were euthanized by intravenously injected overdose of ketamine/xylazine. Mice were dissected, tissues were collected, immediately snap-frozen in liquid nitrogen, and stored at −80 °C. Blood was also collected at culling and plasma ^3^H and ^14^C radioactivity was determined in deproteinized plasma before and after ^3^H_2_O evaporation to estimate glycolysis rates. In hepatic lysates 2-[^14^C] deoxyglucose-6-phosphate was separated from 2-[^14^C] deoxyglucose via ion-exchange columns (Poly-Prep AG1-X8, Bio-Rad, Germany). Glucose uptake rates were calculated by multiplying mean plasma glucose between 120 and 140 min (mmol/ml) by 2-[^14^C] deoxyglucose tissue content (dpm/100 g tissue), divided by 2-[^14^C] deoxyglucose plasma AUC in the same period of time. Source of radioisotopes: Perkin Elmer; samples were measured in Ultima-Gold scintillation cocktail (Tri-Carb2910TR, Perkin-Elmer, Weissenstein, Germany). Whole body glucose disposal (M value) was calculated from the tracer infusion rate, specific activity of [3-^3^H] glucose and body weight.

### Indirect calorimetry

A 48-hours indirect calorimetry trial was conducted in single caged mice having free access to food and water (32-cages PhenoMaster with activity & drinking/feeding monitoring, TSE Systems GmbH, Bad Homburg, Germany). Four data points were collected per hour (oxygen consumption, carbon dioxide production, respiratory exchange ratio, metabolic rate) resulting in a total of 192 data points. From gas exchange data we calculated oxidation rates of carbohydrates and lipids^46^. In parallel to energy expenditure, food consumption was carefully monitored. Food consumption was converted into metabolizable energy uptake using a factor of 13.047 kJ per gram food (Altromin 1324, Altromin, Lage, Germany) that was determined before the indirect calorimetry trial based on bomb calorimetric combustion of diet and feces samples.

### qNMR

The body composition was analyzed by non-invasive qNMR (Bruker MiniSpec LF50, Bruker, Ettlingen, Germany).

### Clinical Chemistry

Blood samples for clinical chemistry analyses were collected in Li-heparin coated tubes after overnight food withdrawal and from *ad libitum* fed mice. Plasma samples were analyzed using an AU480 autoanalyzer (Olympus, Germany) and adapted reagent kits provided by Beckman-Coulter, Wako Chemicals GmbH or Randox according to GMC standard procedures^57^. A small EDTA-blood sample was used by the Immunology screen for FACS analysis.

### FACS Analysis of Peripheral blood leukocytes (PBL)

PBLs were isolated from the cell pellet of 500 μl whole blood samples after centrifugation. The cell pellet is dissolved in 600 μl NH4Cl-based, Tris-buffered erythrocyte lysis solution, and 150 μl transferred into 96-well micro titer plates. After subsequent washing steps with FACS staining buffer (PBS, 0.5% BSA, 0.02%sodium azide, pH 7.45), PBLs were incubated for 20 min with Fc block (clone 2.4G2, PharMingen, San Diego, USA). Cells were then stained with fluorescence-conjugated monoclonal antibodies (PharMingen). After the antibody incubation, propidium iodide was added for the identification of dying/dead cells^45^, which might bind antibodies unspecifically, and/or loose specific antigens upon apoptosis^44^. Samples were acquired from 96 well plates and measured in one of our two threelaser 10-color flow cytometers (LSRII, Becton Dickinson, USA; Gallios, Beckman Coulter, USA). A total number of 10.000–30.000 living CD45+ per sample is reached. For analysis, intact cells are first identified by their FSC/SSC profile. These cells were gated on the basis of their propidium iodide/PE signal (compensated parameters), allowing the dead cells to be gated out. Living cells were then gated using their SSC/CD45 signal, gating out remaining erythrocytes, thrombocytes and debris^58^. CD45+ cells are subsequently analyzed by software based analysis (Flowjo, TreeStar Inc, USA; SPICE^59^. In former experiments, FMO (Fluorescence minus one) controls from wild-type mice have been used to define ‘positive’ and ‘negative’ regions^60^.

### Immunohistochemical analysis

The IHC was performed using the streptavidin-peroxidase method with an automated immunostainer (DiscoveryXT; Roche, Penzberg, Germany) in paraffin-embedded tissue. After heat-induced antigen retrieval with EDTA (pH 8), we used the primary antibody anti-serotonin (5-HT) ABIN617893 from https://www.antibodies-online.com/antibody/617893/anti-5-Hydroxytryptamine in a dilution 1:100. We repeated the procedure two times with an appropriate positive control (neuroendocrine cells in murine gut) and a negative control (without the primary antibody) to confirm the specificity of the staining. Secondary antibody and blocking against nonspecific binding was performed according to antibody information. DAB and hematoxylin as contrast were used for visualization of the reaction peptide-hormone-antibody. Cytoplasmic staining was considered a positive reaction. Images for illustration were made by the slide-scanning system, NanoZoomer 2.0 HT (Hamamatsu Photonics K.K.; Hamamatsu City, Japan).

### DEXA

After anesthesia, the weight and length of the mouse were recorded, and the mouse was placed in the analyzer (pDEXA Sabre X-ray Bone Densitometer, Norland Medical Systems Inc., Basingstoke, Hampshire, UK). Whole body analysis excluding the skull was performed (Scan speed 20 mm/s, Resolution 0.5 mm × 1.0 mm, HAW 0.020).

### Histopathological analysis

The tissues were isolated from the animals, washed in PBS and fixed in formalin at 4 °C overnight. After that they were embedded into paraffin, cut with a microtome and stained with either Hematoxylin/Eosin (H/E) or Periodic acid-Schiff (PAS). Photos were taken with a Nikon Eclipse E800 microscope equipped with a Q Imaging ExiAqua camera using the Bioquant Q-capture Pro7 program. Adipocyte size was calculated using the Adiposoft program.

### Transcriptome analyses

Transcriptome analyses from liver samples of four transgenic and four wildtype male mice at the age of 21 weeks were performed following total RNA extraction (RNAeasy Midi kit, Qiagen). Illumina Mouse Ref-8 v2.0 Expression BeadChips were employed as previously described^61^. Illumina GenomeStudio 2011.1 was used for data normalization (cubic spline) and statistical analysis for the identification of differential gene expression was performed with SAM (Significance Analysis of Microarrays, fold change >1.7, FDR < 1%)^62^. Over-represented functional annotations were obtained using Qiagen’s Ingenuity Pathway Analysis (Qiagen Redwood City). The data discussed in this publication have been deposited in NCBI’s Gene Expression Omnibus^63^ and are accessible through GEO Series accession number GSE95345.

### Identification of the insertion site

The identification of the insertion site was performed by the Cergentis company (Utrecht, Netherlands) using a TLA (Targeted Locus Amplification) sequencing technique^36^.

### Human Growth Hormone and mouse IGF-1 detection

Human Growth Hormone (hGH) was detected and quantified in the serum of TgC6hp55 mice using a Growth Hormone (GH) Human SimpleStep ELISA Kit from Abcam (ab190811). Serum from wt mice did not give any signal. Mouse IGF-1 was detected and quantified in the serum of wt and TgC6hp55 mice using an IGF-1 (mouse/rat) ELISA from ALPCO (Cat. No. 22-IG1MS-E01).

### Treatment with Pegvisomant

Pegvisomant was kindly provided by Pfizer (New York, USA).

Starting at 20 days of age, the mice were first weighed and then injected intraperitoneally every Monday-Wednesday-Friday morning with either 250 mg/kg Pegvisomant (experimental groups) or saline (control groups) according to Liao *et al*.^64^ for 4 weeks. After the end of this period the mice were sacrificed, their organs were weighed and liver and abdominal white adipose tissue were collected for histopathological examination. Additionally, blood serum was collected in order to measure mouse IGF-1 as an indicator of the effectiveness of the treatment.

### Statistical analysis

In every comparison we made, we first examined if the data in each group follow a Gaussian distribution using D’Agostino-Pearson Omnibus normality test, Shapiro-Wilk test and Kolmogorov-Smirnov test with Dallal-Wilkison-Lilliefor P value. If the data indeed followed a Gaussian distribution, we compared them using unpaired t-test or unpaired t-test with Welch’s correction if the variances of the different groups compared were significantly different according to the F-test. On the contrary, if the data were not normally distributed, we compared them using the Mann-Whitney test. Finally, for multiple statistical comparisons on a single data set we used the ANOVA test. Significance is shown in graphs as *p < 0.05, **p < 0.01, ***p < 0.001.

## Electronic supplementary material

Supplementary PDF File
