# Supplementary material for: Extensive phenotypic characterization of a new transgenic mouse reveals pleiotropic perturbations in physiology due to mesenchymal hGH minigene expression
Source: Sci Rep. 2017 May 25;7:2397. doi: 10.1038/s41598-017-02581-8 (PMC5445072; doi:10.1038/s41598-017-02581-8)
Supplement: Supplementary file 1 — Supplementary PDF File [file 41598_2017_2581_MOESM1_ESM.pdf]

Extensive phenotypic characterization of a new transgenic mouse reveals pleiotropic perturbations in physiology due to mesenchymal *hGH* minigene expression.

Aimilios Kaklamanos[1,+], Jan Rozman[4,5,+], Manolis Roulis[1,#], Niki Karagianni[2], Maria Armaka[1], Moya Wu[4], Laura Brachthäuser[4,7], Julia Calzada-Wack[4], Marion Horsch[4], Johannes Beckers[4,5,8], Birgit Rathkolb[4,5,6], Thure Adler[4], Frauke Neff[4,7], Eckhard Wolf[6,5], Valerie Gailus-Durner[4], Helmut Fuchs[4], Martin Hrabe de Angelis[4,5,8], George Kollias[1,3,*].

[1]Biomedical Sciences Research Center (B.S.R.C.) "Alexander Fleming", 16672 Vari, Greece
[2]Biomedcode Hellas SA, Vari, Greece
[3]Department of Physiology, Medical School, National and Kapodistrian University of Athens, 11527 Athens, Greece.
[4]German Mouse Clinic, Institute of Experimental Genetics, Helmholtz Zentrum München, German Research Center for Environmental Health GmbH, Ingolstädter Landstrasse 1, 85764 Neuherberg, Germany;
[5]German Center for Diabetes Research (DZD), Ingolstädter Landstraße 1, 85764 Neuherberg,
[6]Ludwig-Maximilians-Universität München, Gene Center, Institute of Molecular Animal Breeding and Biotechnology, Feodor-Lynen Strasse 25, 81377 Munich, Germany
[7]Institute of Pathology , Helmholtz Zentrum München, German Research Center for Environmental Health, Ingolstädter Landstrasse 1, 85764 Neuherberg, Germany
[8]Chair of Experimental Genetics, Center of Life and Food Sciences Weihenstephan, Technische Universität München, Ingolstaedter Landstrasse 1, 85354 Freising-Weihenstephan, Germany
[+]These authors contributed equally to the work.
#Current address: Department of Immunobiology, Yale University School of Medicine, New Haven, CT 06520, USA
*Correspondence and requests for materials should be addressed to: G.K. (kollias@fleming.gr),

Supplementary Figures captions

Supplementary Figure S1. TgC6hp55 mice do not show increased body weight at the first 2 weeks of age.

(A) Body weight comparison at 1 week (n=29 wt and n=22 TgC6hp55 mice). **(B)** Body weight comparison at 11 days (n=41 wt and n=33 TgC6hp55 mice). **(C)** Body weight comparison at 2 weeks (n=41 wt and n=32 TgC6hp55 mice). Data represent mean±SEM

Supplementary Figure S2. (A) Whole body composition analysis using Nuclear magnetic Resonance (NMR). Measurement of whole body lean and fat mass in wt (n=30) and TgC6hp55 (n=29) mice at the age of 13 weeks. **(B)** Normalization of the NMR values presented in (S2A Fig.) according to body weight. Data represent mean±SEM

Supplementary Figure S3. TgC6hp55 mice show increased energy consumption as well as increased metabolism.

(A) Daily metabolizable energy uptake (N= 15 WT and 11 TgC6hp55 mice). **(B)** Daily energy expenditure (N= 15 WT and 11 TgC6hp55 mice). Data represent mean±SEM

Supplementary Figure S4. TgC6hp55 mice show alterations in bone parameters and metabolism.

(A) Comparison of Bone Mineral Density in wt (n=20) and TgC6hp55 (n=18) mice. **(B)** Comparison of the total bone mass (without calculating the scull) in wt (n=20) and TgC6hp55 (n=18) mice. **(C)** Comparison of the serum Alkaline Phosphatase activity in wt (n=29) and

TgC6hp55 (n=30) mice. Data represent mean±SEM

Supplementary Figure S5. TgC6hp55 mice show alterations in a plethora of parameters of their physiology.

(A-F) Hematological analysis of the serum of wt (n=28) and TgC6hp55 (n=28) mice: **(A)** Comparison of Hematocrit. **(B)** Comparison of Hemoglobin. **(C)** Comparison of Mean Corpuscular Volume (MCV). **(D)** Comparison of Mean Corpuscular Hemoglobin (MCH). **(E)** Comparison of Red Blood Cell Distribution Width (RDW). **(F)** Comparison of White Blood Cell numbers. **(G-L)** Biochemical analysis of the serum of wt (n=29) and TgC6hp55 (n=30) mice: **(G)** Comparison of Iron concentration in the plasma. **(H)** Comparison of the Total Iron Binding Capacity (TIBC). **(I)** Comparison of the calculated transferrin saturation. **(J)** Comparison of the total serum protein. **(K)** Comparison of the serum Albumin levels. **(L)** Comparison of serum triglycerides in ad libitum fed mice. **(M-N)** Immunological analysis in the peripheral blood of wt and TgC6hp55 mice: **(M)** Comparison of $LyC6^{+}$ monocytes from wt (n=22) and TgC6hp55 (n=23) mice. **(N)** Comparison of $CD4^{+}CD45^{+}CD44^{++}$ T cells from wt (n=29) and TgC6hp55 (n=30) mice. **(O-P)** Immunological analysis in the spleen of wt (n=4) and TgC6hp55 (n=4) mice: **(O)** Comparison of $B220^{+}$ cells. **(P)** Comparison of $Mac1^{+}Gr1^{lo}$ cells. Data represent mean±SEM

Supplementary Figure S6. TgC6hp55*Tnf* $^{-/-}$ and TgC6hp55*Tradd*$^{D/D}$ mice show increased organ weights similar to the TgC6hp55 mice.

(A) Comparison of liver, abdominal white adipose tissue (WAT), lung (both lungs), kidney (both kidneys) and spleen weight among *Tnf*$^{+/+}$ (n=6), *Tnf* $^{-/-}$(n=11), TgC6hp55*Tnf*$^{+/+}$ (n=6) and TgC6hp55*Tnf* $^{-/-}$ (n=9) mice. **(B)** Comparison of liver, abdominal white adipose tissue (WAT), lung (both lungs), kidney (both kidneys) and spleen weight among *Tradd*$^{+/+}$ (n=8), *Tradd* $^{D/D}$

(n=7), TgC6hp55*Tradd*$^{+/+}$ (n=7) and TgC6hp55*Tradd* $^{D/D}$ (n=7) mice. **(C)** Comparison of the normalized organ weights from (S6A Fig) expressed as % of the mouse body weight. **(D)** Comparison of the normalized organ weights from (S6B Fig) expressed as % of the mouse body weight. Data represent mean±SEM

Supplementary Figure S7. TgC6hp55*Tnf*$^{-/-}$ and TgC6hp55*Tradd*$^{D/D}$ mice show histopathological alterations in their liver and white adipose tissue similar to the TgC6hp55 mice.

(A) Histopathological assessment of lipid accumulation in the livers of *Tnf* $^{+/+}$, *Tnf* $^{-/-}$, TgC6hp55*Tnf*$^{+/+}$and TgC6hp55*Tnf* $^{-/-}$mice (H/E staining). **(B)** Histopathological comparison of the adipocyte size from the abdominal white adipose tissue of *Tnf* $^{+/+}$, *Tnf* $^{-/-}$, TgC6hp55*Tnf*$^{+/+}$and TgC6hp55*Tnf* $^{-/-}$mice (H/E staining). **(C)** Histopathological assessment of lipid accumulation in the livers of *Tradd*$^{-+/+}$, *Tradd*$^{D/D}$, TgC6hp55*Tradd*$^{+/+}$and TgC6hp55*Tradd*$^{D/D}$ mice (H/E staining). **(D)** Histopathological comparison of the adipocyte size from the abdominal white adipose tissue of *Tradd*$^{-+/+}$, *Tradd*$^{D/D}$, TgC6hp55*Tradd*$^{+/+}$and TgC6hp55*Tradd*$^{D/D}$ mice (H/E staining). *Tnf*$^{+/+}$ (n=6), *Tnf* $^{-/-}$(n=11), TgC6hp55*Tnf*$^{+/+}$ (n=6) and TgC6hp55*Tnf* $^{-/-}$ (n=9), *Tradd*$^{+/+}$ (n=8), *Tradd* $^{D/D}$ (n=7), TgC6hp55*Tradd*$^{+/+}$ (n=7) and TgC6hp55*Tradd* $^{D/D}$ (n=7) mice. All figures are in 200x magnification / scale=60μm.

Supplementary Figure S8. TgC6hp55 mice coming from Founder 2 keep showing enhanced somatic growth identical to that observed in the mice coming from Founder 1. Body weight curve in wt mice coming from Founder 1 (n=10), transgenic mice coming from Founder 1 (n=10), wt mice coming from Founder 2 (n=11) and transgenic mice coming from Founder 2

(n=15). At all time points, wt mice from both Founders do not show any difference in their body weight. Simultaneously, the transgenic mice coming from both Founders also show no difference in their body weight at all time points.

a represents *** statistical difference between the body weight of TgC6hp55 mice coming from Founder 1 and the body weight of the wt mice coming from Founder 1. **b** represents ** statistical difference between the body weight of the transgenic mice coming from Founder 2 and the body weight of the wt mice coming from Founder 2. **c** represents *** statistical difference between the body weight of the transgenic mice coming from Founder 2 and the body weight of the wt mice coming from Founder 2. Data represent mean±SEM

Supplementary Figure S9. TgC6hp55 mice show enhanced production of serotonin in their pancreatic islets.

A) Representative serotonin immunostaining (brown) of neuroendocrine pancreas (islets) of a TgC6hp55 mouse and a wildtype mouse. B) The negative controls of the immunostaining in the respective tissue (without the primary antibody). n=3 male and 3 female wild type mice and n=3 male and 3 female TgC6hp55 mice. All figures are in 400x magnification / scale = 50μm.

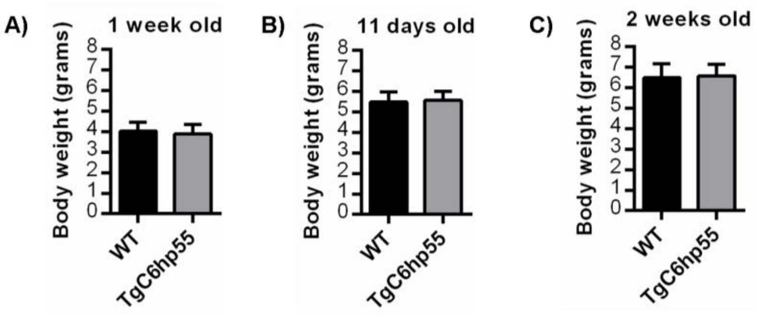

Supplementary Figure S1

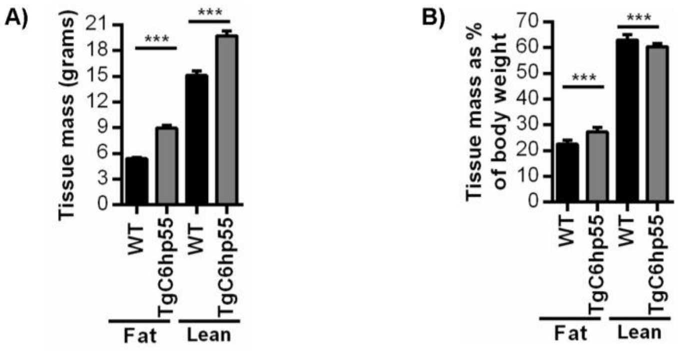

Supplementary Figure S2

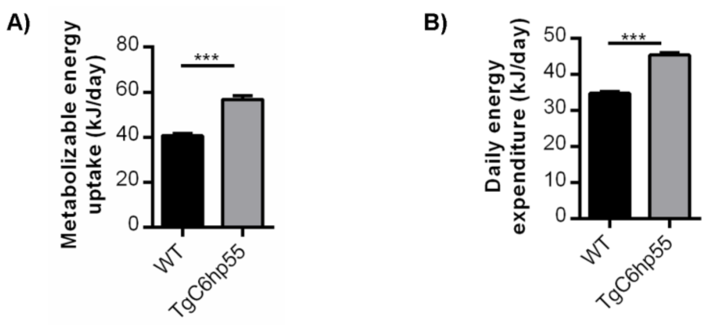

Supplementary Figure S3

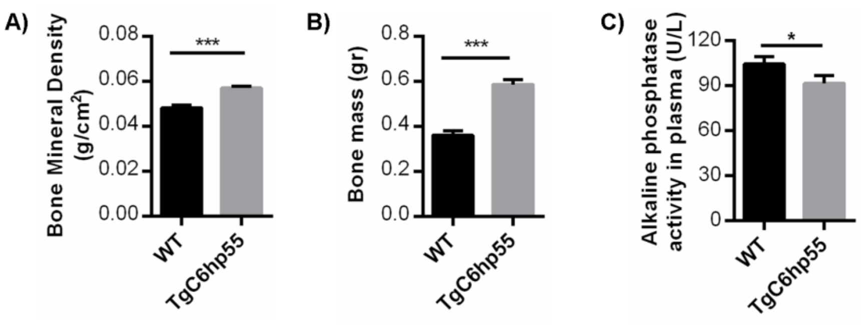

Supplementary Figure S4

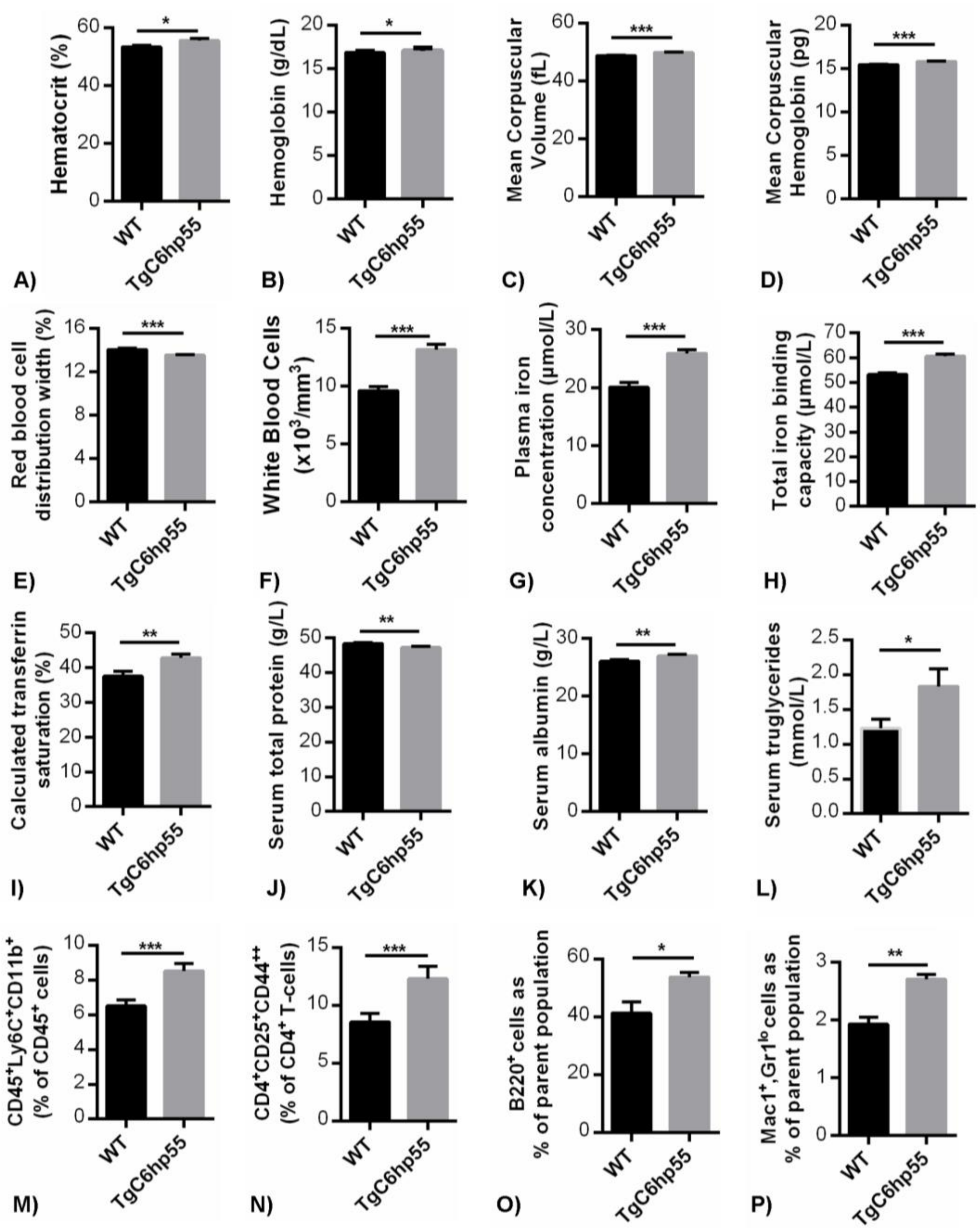

Supplementary Figure S5

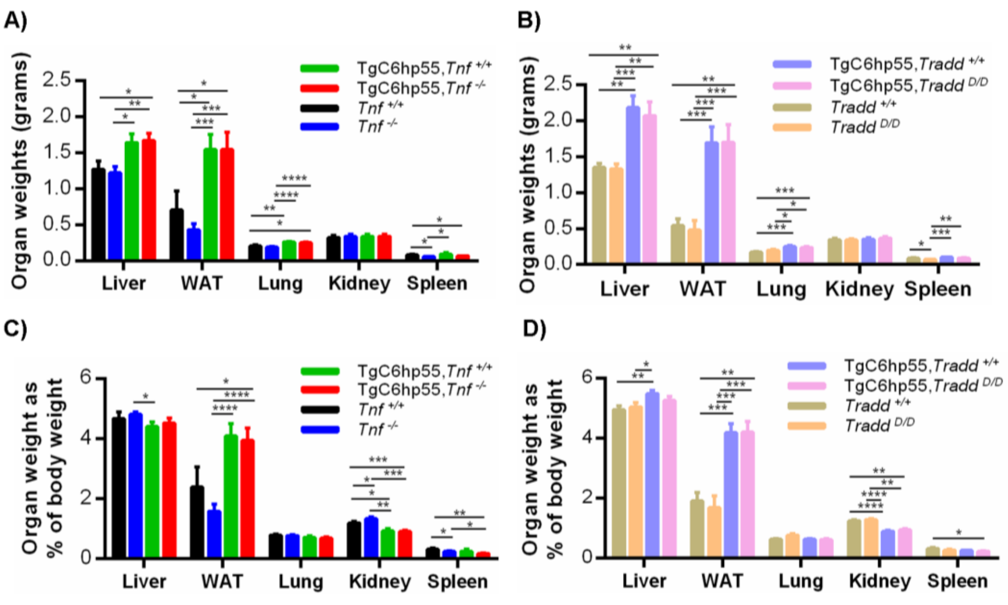

Supplementary Figure S6

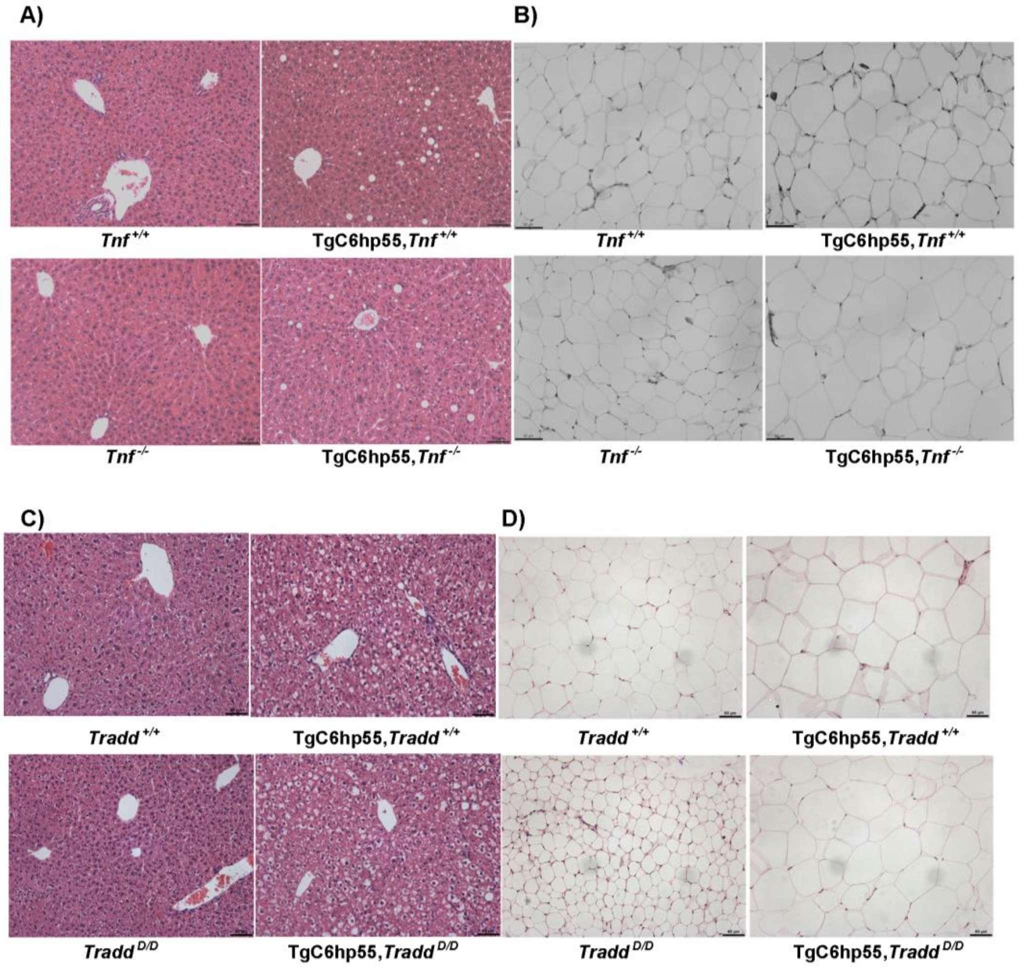

Supplementary Figure S7

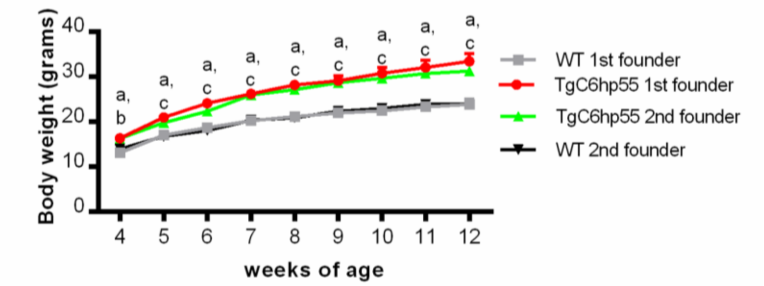

Supplementary Figure S8

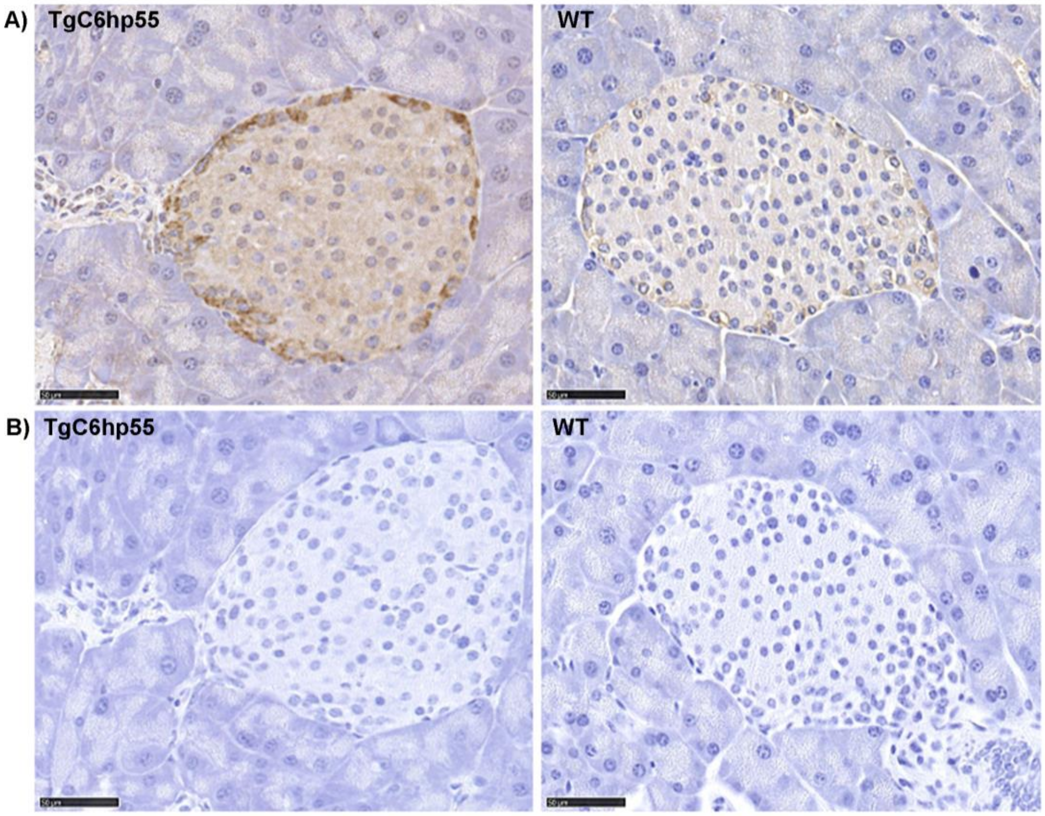

Supplementary Figure S9
